# Effectiveness of One-Way Text Messaging on Attendance to Follow-Up Cervical Cancer Screening Among Human Papillomavirus–Positive Tanzanian Women (Connected2Care): Parallel-Group Randomized Controlled Trial

**DOI:** 10.2196/15863

**Published:** 2020-04-02

**Authors:** Ditte S Linde, Marianne S Andersen, Julius Mwaiselage, Rachel Manongi, Susanne K Kjaer, Vibeke Rasch

**Affiliations:** 1 Department of Clinical Research University of Southern Denmark Odense Denmark; 2 Department of Obstetrics and Gynaecology Odense University Hospital Odense Denmark; 3 Odense Patient Data Explorative Network Odense University Hospital Odense Denmark; 4 Department of Medical Endocrinology Odense University Hospital Odense Denmark; 5 Department for Cancer Prevention Services Ocean Road Cancer Institute Dar es Salaam United Republic of Tanzania; 6 Department of Public Health Kilimanjaro Christian Medical University College Moshi United Republic of Tanzania; 7 Department of Gynaecology Rigshospitalet University Hospital Copenhagen Denmark; 8 Department of Virus, Lifestyle and Genes Danish Cancer Society Research Center Copenhagen Denmark

**Keywords:** telemedicine, cervical cancer, HPV, early detection of cancer, Africa

## Abstract

**Background:**

Rapid human papillomavirus (HPV) DNA testing is an emerging cervical cancer screening strategy in resource-limited countries, yet it requires follow-up of women who test HPV positive.

**Objective:**

This study aimed to determine if one-way text messages improved attendance to a 14-month follow-up cervical cancer screening among HPV-positive women.

**Methods:**

This multicenter, parallel-group randomized controlled trial was conducted at 3 hospitals in Tanzania. Eligible participants were aged between 25 and 60 years, had tested positive to a rapid HPV test during a patient-initiated screening, had been informed of their HPV result, and had a private mobile phone with a valid number. Participants were randomly assigned in a 1:1 ratio to the intervention or control group through an incorporated algorithm in the text message system. The intervention group received one-way text messages, and the control group received no text messages. The primary outcome was attendance at a 14-month health provider-initiated follow-up screening. Participants were not blinded, but outcome assessors were. The analysis was based on intention to treat.

**Results:**

Between August 2015 and July 2017, 4080 women were screened for cervical cancer, of which 705 were included in this trial—358 women were allocated to the intervention group, and 347 women were allocated to the control group. Moreover, 16 women were excluded before the analysis because they developed cervical cancer or died (8 from each group). In the intervention group, 24.0% (84/350) women attended their follow-up screening, and in the control group, 23.8% (80/335) women attended their follow-up screening (risk ratio 1.02, 95% CI 0.79-1.33).

**Conclusions:**

Attendance to a health provider-initiated follow-up cervical cancer screening among HPV-positive women was strikingly low, and one-way text messages did not improve the attendance rate. Implementation of rapid HPV testing as a primary screening method at the clinic level entails the challenge of ensuring a proper follow-up of women.

**Trial Registration:**

ClinicalTrials.gov NCT02509702; https://clinicaltrials.gov/ct2/show/NCT02509702.

**International Registered Report Identifier (IRRID):**

RR2-10.2196/10.2196/15863

## Introduction

### Background

Despite the fact that cervical cancer can be prevented, the global age-standardized incidence and mortality rates of cervical cancer were 13.1 and 6.9, respectively, per 100,000 women in 2018 [1]. Cervical cancer is the fourth most common cancer among women worldwide [1], and the burden of disease is unequally distributed, with 70% of the cases occurring in low-resource countries [2]. In Tanzania, cervical cancer constitutes 38% of all new cancer cases among women, and the age-standardized incidence and mortality rates were 59.1 and 42.7, respectively, per 100,000 women in 2018 [3].

Primary prevention of cervical cancer includes vaccination against high-risk human papillomavirus (HPV) types, and secondary prevention involves early and accurate detection through screening and subsequent treatment of precancerous lesions [4]. In 2018, Tanzania launched HPV vaccination of girls aged 9 to 14 years [5]; however, older generations of women fully rely on screening to prevent the development of cervical cancer. Currently, Tanzania follows the World Health Organization’s *screen-and-treat* guideline for resource-limited settings, which involves visual inspection with acetic acid (VIA) with immediate treatment if lesions appear [6]. Rapid HPV DNA testing is an emerging screening strategy in resource-limited countries, as it has proven to be an objective and more sensitive alternative to VIA [7,8]. However, the test only identifies women who are high-risk HPV positive and thus at increased risk of developing cervical cancer. Consequently, subsequent screening of these women is needed to identify those who have precancerous lesions. This leads to a risk of loss to follow-up compared with screening by the use of VIA. Yet, this issue is still largely unexplored in an African context.

In recent years, mobile phone access has expanded rapidly across Africa, and in 2018, there were 81 mobile subscriptions per 100 people in Tanzania [9]. The increasing mobile phone access has gone hand in hand with a growing number of mobile health (mHealth) interventions that aim to address global health issues in innovative ways. One-way text message interventions—also referred to as one-way SMS—involve sending text messages to a recipient who cannot respond to the text message. The advantages of text messages are that they are easy to use, can be sent to the receivers simultaneously, and require less staff. A recent systematic review and meta-analysis of one-way text message trials in Africa showed that one-way text messages have been tested within different clinical areas across Africa, although mainly in relation to medicine adherence and appointment attendance. The effect of one-way text messages varied across clinical areas, and overall, the highest effect was found in relation to increasing attendance to a childhood immunization appointment [10]. In relation to cervical cancer screening attendance, a systematic review from 2017 concerning text message interventions on cancer screening rates [11] found 1 trial from Malaysia with no effect of one-way text messages improving attendance to a repeat cervical smear compared with postal letters [12]. However, recent trials from Tanzania and Kenya have shown that one-way text messages increased attendance to cervical cancer screening among screening-naïve women [13] and a repeat cervical smear [14], compared with no text messages. Yet, it is still unknown whether or not one-way text messages can increase the attendance rate of HPV-positive women who have been appointed a follow-up screening.

### Objectives and Study Context

The aim of this study (*Connected2Care)* was to assess the effect of one-way text messages on attendance to a provider-initiated follow-up screening appointment among women who had tested HPV positive during a patient-initiated opportunistic screening 14 months earlier. In addition, we examined factors associated with attendance regardless of group allocation. *Connected2Care* is a substudy of a larger research study, Comprehensive Cervical Cancer Prevention in Tanzania (CONCEPT). The CONCEPT study is linked to the existing national cervical cancer screening programs in Dar es Salaam and Kilimanjaro and will end in December 2021.

## Methods

### Study Design

*Connected2Care* is an unblinded, multicenter, parallel-group randomized controlled trial conducted at 3 hospitals in Tanzania: Ocean Road Cancer Institute (ORCI) in Dar es Salaam and Kilimanjaro Christian Medical Centre (KCMC) and Mawenzi Regional Hospital in Moshi. Originally, the study was designed as a double-site study (ORCI and KCMC); however, because of a slower-than-expected recruitment rate, a third study site (Mawenzi Regional Hospital) was added 6 months into the recruitment phase. To increase recruitment further, fliers informing about the screening were shared at mosques and churches close to the study sites, and 2 outreach screenings were conducted by the ORCI screening nurses in Dar es Salaam. The study protocol has been published elsewhere [15], and the joined ethical approval for all study sites was obtained from the National Institute for Medical Research in Tanzania.

### Participants

Women aged 25 to 60 years, who attended a patient-initiated cervical cancer screening at the study sites, were assessed by a screening nurse for overall eligibility to participate in the CONCEPT study. Eligible women gave written informed consent. The exclusion criteria were pregnancy or menstruation on the day of enrollment, previous hysterectomy, cervical cancer, or diagnosis of cervical precancerous lesions within the past 12 months. The subgroup of CONCEPT participants, who tested high-risk HPV positive at the enrollment screening, was assessed for eligibility to be further included in the *Connected2Care* study. Women were ineligible for inclusion in *Connected2Care* if they did not own a private mobile phone, had provided an invalid phone number (ie, digits missing), or were not informed of their positive HPV test result. Ineligible women were excluded before randomization.

### Randomization and Blinding

Participants were randomly assigned to the intervention or control group in a 1:1 ratio. The randomization occurred through an incorporated algorithm in the text message system, which automatically assigned participants to the intervention or control group. The random sequence generator was developed by the external information technology consultant, who developed the text messaging system, and it was concealed to all investigators. Screening nurses enrolled participants and were concealed to the latter group allocation. The first author (DL) assigned participants to the trial by uploading the phone numbers of the eligible participants into the text messaging system and was not concealed to the group allocation. Due to the overt nature of the text message intervention, the study participants were not concealed to their group allocation. Yet, the outcome assessors—in the form of screening nurses registering the attendance date—were blinded.

### Procedures

Eligible women were assigned an anonymous study ID and interviewed by a trained nurse using a structured questionnaire. The nurse also registered their home address and phone numbers as well as provided general cervical cancer screening education and counseling according to the national guidelines for cervical cancer screening in Tanzania. During the enrollment screening, 2 cervical specimens were taken using a careHPV brush [16] and a ThinPrep Pap Test plastic spatula [17] before VIA was conducted. Women who tested VIA positive were treated onsite with cryotherapy or loop electrosurgical excision procedure. VIA status and treatment did not influence inclusion into the trial, and the results from the ThinPrep-collected specimens (cytology and hybrid capture 2) were not available until postinclusion, hence did not influence enrollment either. The test performance of VIA, careHPV, and Hybrid Capture2 for the detection of cytologically diagnosed high-grade cervical lesions or cancer is not addressed in the *Connected2Care* trial, but in other parts of the CONCEPT project [18]. Once the screening had finished, all women were assigned a 14-month follow-up screening appointment, which was written on a physical appointment card together with their study ID. The appointment card was considered standard care.

Using the rapid careHPV test, the specimens were tested for 14 types of high-risk HPV (16, 18, 31, 33, 35, 39, 45, 51, 52, 56, 58, 59, 66, and 68) in a local laboratory at 2 of the study sites (ORCI and KCMC). Community nurses attempted to conduct a private home visit to inform all careHPV-positive women about their results. The women were informed that 1 of the extra tests they had had during their cervical cancer screening (HPV test) turned out positive, yet this did not mean that they had cervical cancer. Furthermore, they were informed that HPV is a common infection that most likely will clear on its own, but it was important that they attended their follow-up screening as it can develop into cervical cancer over time. Finally, the women were told that they may receive health educative and reminder text messages before their next screening appointment. Inclusion into *Connected2Care* did not occur until it was confirmed to the first author (DL) that a woman had been informed of her result, after which she was uploaded to the text message system and assigned to either the *text message plus standard care group* (intervention group) or the *standard care group* (control group).

**Figure 1:**
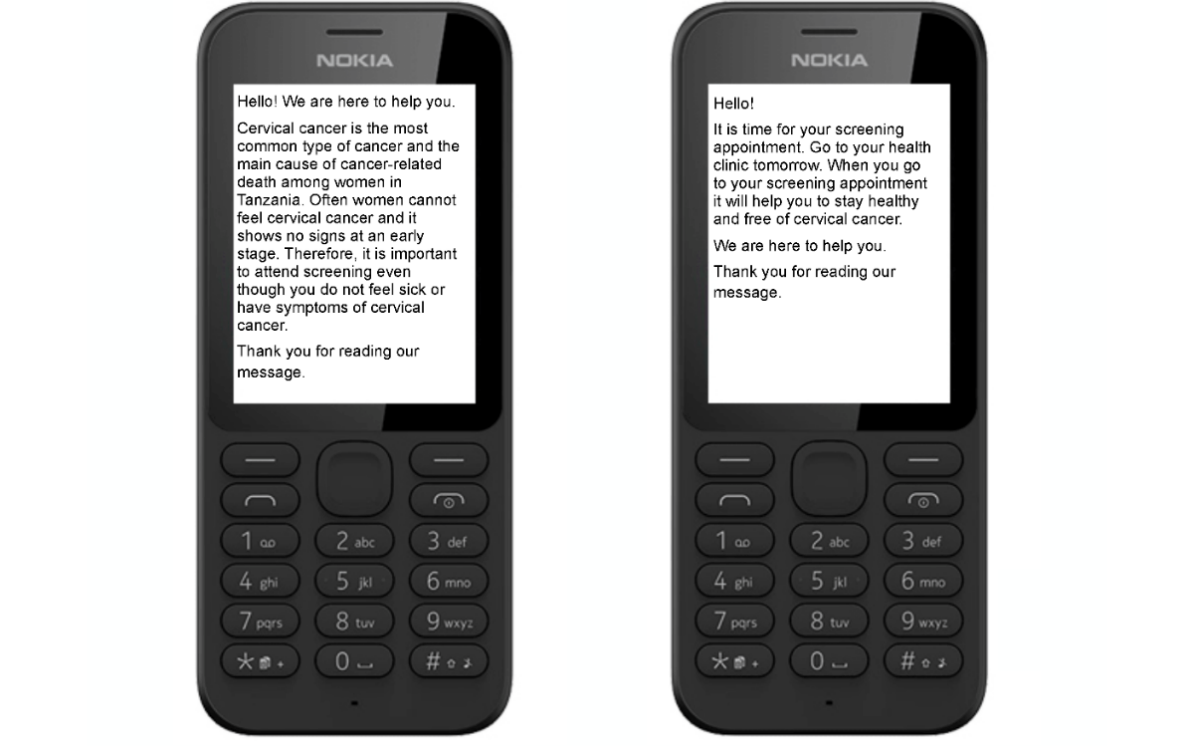
Example of intervention text messages.

Over a period of 10 months, 10 health educative messages, and 5 reminders were sent to the women in the intervention group. The health educative messages were sent once a month and contained information about screening, risk factors, and symptoms for cervical cancer (Figure 1). The reminders were sent 14, 7, and 1 day before the 14-month follow-up screening appointment as well as 1 and 7 days post the appointment date. The sender ID, content, timing, and number of text messages were pretested on screening clients as well as on screening nurses before starting the intervention. The rationale behind combining health education and reminders was that health education would enhance the perceived seriousness of the disease as well as the benefit of the screening, and combined with reminders, they would be cues to actions for screening attendance. The messages were not personalized out of privacy concerns, and they were developed in English and translated into Kiswahili using back-and-forth translation. A Web-based text message portal [19]—developed specifically for this study—automatically dispatched the messages and sent them with the ID *Elimu Ya Afya* (meaning *health education* in Kiswahili). The portal had a *delivery note feature*, which showed the discrepancy between the number of text messages sent and the number of text messages received. All data were entered and stored in Research Electronic Data Capture [20].

The women who attended their follow-up screening had a cervical specimen collected using the ThinPrep Pap Test plastic spatula and went through a standard VIA screening. Furthermore, they were reinterviewed using a structured questionnaire. Their attendance date was noted down in a registration book by the screening nurse. If a woman did not attend her follow-up screening within 30 days of their appointment date, the *Connected2Care* trial was considered finished, and an *active posttrial follow-up* started to trace the nonattending woman. First, a nurse called the woman to encourage her to attend her appointment at the clinic and offered to refund transportation costs. Second, if the woman did not attend, a nurse visited her at home and encouraged her to come to the clinic and attend her appointment. Third, if the woman still did not attend and she had consented to it, an outreach home visit was conducted, where the woman was interviewed, and a self-collected cervical swab was collected using the Evalyn brush [21]. This swab substituted the swab that would have been collected at the screening clinic by the use of the ThinPrep Pap Test plastic spatula, and these results are yet to be published as another part of the CONCEPT study.

### Outcomes

The primary outcome was attendance at a health provider–initiated follow-up screening at 14 months. It was measured as whether or not the participants attended the follow-up screening within 30 days of their screening appointment. Post hoc, we exploratively assessed factors that were associated with attendance, that is, the number of text messages received and sociodemographic characteristics, as well as how attendance had increased postintervention via phone calls and home visits. Prespecified secondary outcomes included cost-effectiveness of the intervention, the intervention’s effect on the knowledge of cervical cancer and screening (16-item true-false questionnaire), and barriers against implementing a text message intervention in Tanzania (mixed methods study). The secondary outcomes, cost-effectiveness and knowledge, have not been assessed, and the barriers against implementation have been assessed in a qualitative postintervention study together with factors that influence screening attendance. The latter objective has been published elsewhere [22], whereas data on barriers related to the text message intervention are yet to be published.

### Statistical Analysis

The sample size was calculated based on the methods described by Altman [23]. We estimated that the intervention would increase the attendance rate with 15 points and that 73% of women in the intervention group, and 58% of women in the control group would attend their follow-up appointment. With an allocation ratio of 1:1, a two-sided alpha of 5%, and 80% power, we required approximately 350 participants in each arm. At the time of design, no other text message interventions on cervical cancer had been conducted in Africa, and the estimation was based on the effect of mHealth interventions found within other clinical areas in Eastern Africa [24,25]. The analysis was based on intention to treat, and participants who developed cervical cancer or died during the intervention period were excluded before analysis, as attendance was not an option for them. We calculated a risk ratio (RR) to determine if the proportion of participants who attended their follow-up screening differed between groups and used a relative risk regression by the use of a generalized linear model with log-link function and binomial distribution as statistical family. In subgroup analyses, we assessed if the intervention had a differential effect across subgroups by including an interaction term between the intervention allocation and a subgroup-defining variable. Stata 15 [26] was used to analyze the data. A data monitoring committee did not oversee the study. The study has been registered at ClinicalTrials.gov (NCT02509702).

## Results

### Trial Results

**Figure 2:**
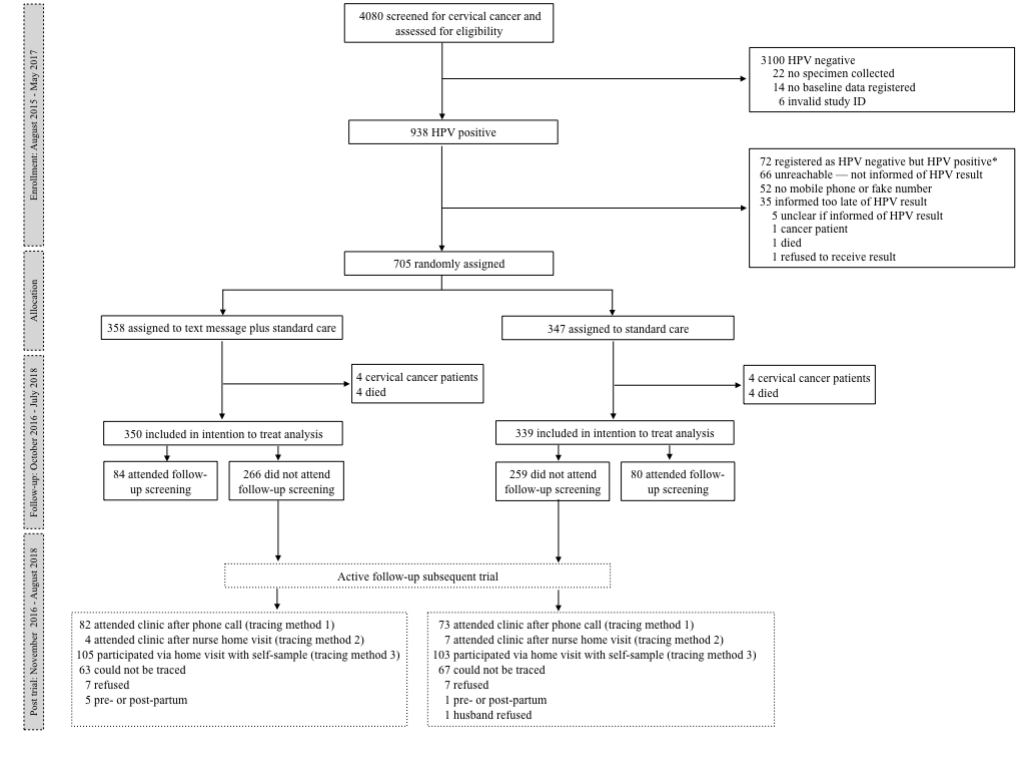
Flowchart of the trial. *Found out post inclusion. HPV: human papillomavirus.

Study participants were enrolled in the study between August 17, 2015, and July 6, 2017, and the follow-up ended on October 6, 2018. Altogether 4080 women were enrolled in the CONCEPT study. Of these women, 705 were included in *Connected2Care* (Figure 2)*.* After randomization, 4 participants in each group developed cervical cancer, and 4 participants from each group died from the disease (n=16). These participants were excluded from the analysis, leaving 689 women for analysis.

The intervention and control group were comparable (Table 1), and the primary analysis showed that the intervention had no effect on attendance to a follow-up screening appointment. Of the 350 women in the intervention group, 84 (24.0%) attended their follow-up screening, and of the 335 women in the control group, 80 (23.8%) attended their follow-up screening (RR 1.02, 95% CI 0.79-1.33).

**Figure 3:**
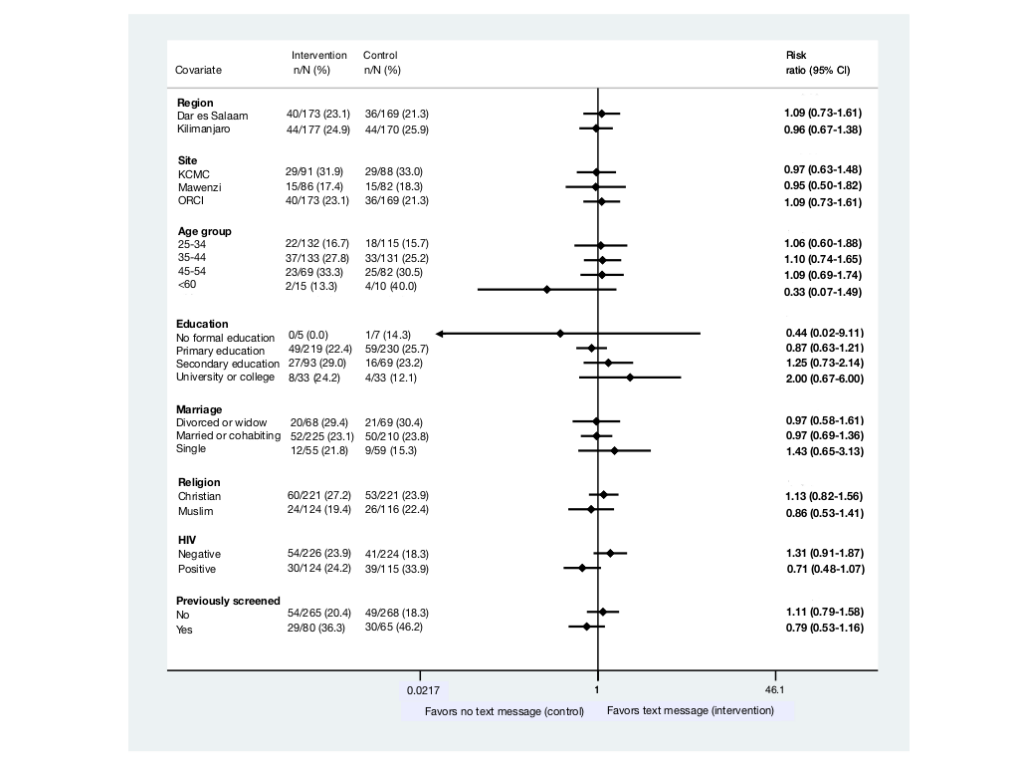
Forrest plot of subgroup analysis. KCMC: Kilimanjaro Christian Medical Centre; ORCI: Ocean Road Cancer Institute.

Six months into the study—after enrollment of 38 participants—we discovered that the SMS system did not dispatch the text messages according to the study plan. We reassigned 38 participants into a new SMS system [19], and they stayed in the groups they were originally allocated to [15]. We conducted a post hoc sensitivity analysis excluding these women and another post hoc analysis excluding 16 participants who were misclassified as HPV positive and erroneously included in the study. The sensitivity analysis of the SMS system (RR 0.95, 95% CI 0.71-1.26) and misclassifications (RR 0.98, 95% CI 0.75-1.29) showed similar results as our main analysis. Moreover, 1 of 8 explorative subgroup analyses showed that HIV status was a potential effect modifier (not adjusted for multiplicity; Figure 3).

**Table 1:** Baseline characteristics.

Sociodemographic characteristics			Text message group (n=350)		Control group (n=339)
**Region, n (%)**					
	Dar es Salaam	173 (49.4)		169 (49.8)	
	Kilimanjaro	177 (50.6)		170 (50.1)	
**Clinic, n (%)**					
	Ocean Road Cancer Institute	173 (49.4)		169 (59.8)	
	Kilimanjaro Christian Medical Centre	91 (26.0)		88 (25.9)	
	Mawenzi Regional Hospital	86 (24.6)		82 (24.2)	
Age (years), mean (SD)			38.2 (8.8)		39.2 (8.4)
**Education, n (%)**					
	No formal education	5 (1.4)		7 (2.1)	
	Primary education	219 (62.6)		230 (67.8)	
	Secondary education	93 (26.6)		69 (20.4)	
	University or college	33 (9.4)		33 (9.7)	
**Marital status^a^, n (%)**					
	Married or cohabiting	226 (64.6)		210 (61.9)	
	Single	55 (15.7)		59 (17.4)	
	Divorced or widow	68 (19.4)		69 (20.1)	
**Religion^a^, n (%)**					
	Christian	223 (63.7)		221 (65.2)	
	Muslim	124 (35.4)		116 (34.2)	
**HIV status, n (%)**					
	HIV positive	124 (35.4)		115 (33.9)	
	HIV negative	226 (64.6)		224 (66.1)	
**Previously screened for cervical cancer^a^, n (%)**					
	Yes	81 (23.1)		65 (19.2)	
	No	268 (77.6)		271 (80.0)	
**Self-perceived health^a^, n (%)**					
	Excellent or very good	62 (17.7)		77 (22.7)	
	Good	239 (68.3)		200 (59.0)	
	Bad or less good	48 (13.7)		60 (17.7)	

^a^Missing under 1%.

The SMS dispatching system showed that all participants in the intervention group received at least one of the 15 text messages sent to them, 8% (2/26) received between 1 and 4 text messages, and 23.4% (26/111) received all 15 text messages (Table 2). When examining factors potentially associated with attendance to screening, we found that the number of text messages received did not appear to affect the attendance rate. In contrast, women aged 35 to 44 years (RR 1.64, 95% CI 1.16-2.33) and 45 to 54 years (RR 1.97, 95% CI 1.36-2.84) were more likely to attend their screening compared with women aged 25 to 34 years; HIV-positive women were more likely to attend their screening than HIV-negative women (RR 1.37, 95% CI 1.05-1.79); and women who had previously been screened were more likely to attend their screening than women who had not been screened before their screening at baseline (RR 2.09, 95% CI 1.61-2.72; Table 2).

**Table 2:** Factors associated with attendance.

Sociodemographic and SMS characteristics		Attendance, n (%)	Attendance (adjusted for SMS; linear)		
			Risk ratio (95% CI)	SE	z-statistic
**SMS received^a^**					
	15 (all, n=111)	26 (23.4)	1.00	N/A^b^	N/A
	10-14 (n=141)	37 (26.2)	1.12 (0.68-1.48)	0.25	0.51
	9-5 (n=65)	18 (28)	1.18 (0.72-1.73)	0.31	0.63
	1-4 (n=26)	2 (8)	0.32 (0.68-1.48)	0.23	−1.59
	0 (control group, n=339)	80 (23.6)	1.00 (0.61-1.67)	0.2	0.04
**Region**					
	Dar es Salaam (n=342)	76 (22.2)	1.00	N/A	N/A
	Kilimanjaro (KCMC^c^/Mawenzi, n=347)	88 (25.3)	1.14 (0.87-1.49)	0.16	0.96
**Clinic**					
	Ocean Road Cancer Institute (n=342)	76 (22.2)	1.00	N/A	N/A
	KCMC (n=179)	58 (32.4)	1.46 (1.09-1.95)	0.22	2.55
	Mawenzi Regional Hospital (n=168)	30 (17.9)	0.80 (0.55-1.18)	0.16	−1.13
**Age (years)^d^**					
	25-34 (n=248)	40 (16.1)	1.00	N/A	N/A
	35-44 (n=264)	70 (26.5)	1.64 (1.16-2.33)	0.29	2.8
	45-54 (n=151)	48 (31.8)	1.97 (1.36-2.84)	1.97	0.37
	55-60 (n=26)	6 (23)	1.47 (0.69-3.13)	1.47	0.57
**Education**					
	No formal education (n=12)	1 (8)	0.35 (0.53-2.28)	0.33	−1.10
	Primary education (n=449)	108 (24.1)	1.00	N/A	N/A
	Secondary education (n=162)	43 (26.5)	1.10 (0.81-1.50)	0.17	0.63
	University or college (n=66)	12 (18)	0.76 (0.44-1.29)	0.76	0.21
**Marital status^e^**					
	Married or cohabiting (n=436)	102 (23.4)	1.00	N/A	N/A
	Single (n=114)	21 (18.4)	0.79 (0.52-1.20)	0.17	−1.12
	Divorced or widow (n=137)	41 (29.9)	1.28 (0.94-1.74)	0.2	1.56
**Religion^e^**					
	Christian (n=444)	113 (25.5)	1.00	N/A	N/A
	Muslim (n=240)	50 (20.8)	0.81 (0.61-1.09)	0.12	−1.37
**HIV status**					
	HIV negative (n=450)	95 (21.1)	1.00	N/A	N/A
	HIV positive (n=239)	69 (28.0)	1.37 (1.05-1.79)	0.19	2.29
**Previously screened for cervical cancer^e^**					
	No (n=539)	104 (19.3)	1.00	N/A	N/A
	Yes (n=146)	59 (40.4)	2.11 (1.62-2.75)	0.28	5.57
**Self-perceived health^e^**					
	Excellent or very good (n=139)	31 (22.3)	1.00	N/A	N/A
	Good (n=439)	108 (24.6)	1.10 (0.78-1.57)	0.2	0.56
	Bad or less good (n=108)	25 (23.1)	1.04 (0.65-1.65)	0.25	0.16

^a^For 7 participants, it is unknown how many text messages were received. Not adjusted for SMS linear.

^b^Not applicable.

^c^KCMC: Kilimanjaro Christian Medical Centre.

^d^Risk Ratio, continuous (95% CI): 1.03 (1.01-1.04).

^e^Missing under 1%.

### Posttrial Results

Once the trial had finished, the *active* posttrial follow-up started where women in the intervention and control groups were traced using the same methods. Overall, 24% of women attended their screening at the clinic within 30 days of their appointment (trial period). An additional 23% of women attended the follow-up screening at the clinic after being called (tracing method 1), and a further 2% of women attended the clinic after having a nurse home visit (tracing method 2). Finally, an additional 30% of women had their follow-up screening via a self-sample test during a home visit (tracing method 3). Hence, the total number of women who received a follow-up screening was 77.9% (537/689; Table 3).

**Table 3:** Post follow-up attendance rate.

Attendance		Text message group (n=350), n (%)		Control group (n=339), n (%)		Total (text message+control group combined, n=689), n (%)	
**During trial**							
	Attendance, primary analysis		84 (24.0)		80 (23.4)		164 (24.4)
**Post trial—both groups receive the same tracing methods**							
	Attendance at clinic after a phone call (tracing method 1)		82 (23.4)		73 (21.5)		155 (22.5)
	Attendance at clinic after a home visit (tracing method 2)		4 (1.1)		7 (2.1)		11 (1.6)
	Participation via self-sampling at the home level (tracing method 3)		105 (30.0)		103 (30.3)		208 (30.1)
Total		275 (78.5)		262 (77.2)		537 (77.9)	

## Discussion

### Principal Findings

This trial shows that one-way text messages did not improve the attendance rate of a health provider-initiated follow-up cervical cancer screening among HPV-positive women. Overall, one-fourth of the participants attended their follow-up screening regardless of whether or not they had received text messages. Hence, the barrier of getting women to return to the clinic was not one that one-way text messages appeared to overcome. Not all text messages dispatched were received among the intervention group, yet the number of messages received did not affect the attendance rate. Once the trial had finished, an additional 23% of women attended the clinic after a nurse had called them, and 2% of women attended after a nurse home visit. Of the remaining nonattendees, a further 30% of women participated via a home visit and self-sampling, which led to a total follow-up rate of 78%.

### Limitations

We encountered several obstacles while conducting this trial. First, we had to switch to a new SMS system 6 months into the trial because of the first system being unreliable. However, our sensitivity analysis found no difference in effect among the participants who had been enrolled using the first system. Second, a number of women were misclassified as HPV negative or HPV positive in the process of transferring the laboratory reports to the CONCEPT investigators. This led to a number of women being incorrectly excluded from or included in the trial. The sensitivity analysis of the incorrectly included women showed no difference in results. Although these issues are study specific, they highlight a need for incorporating secure procedures when implementing a more complex screening method such as rapid HPV testing in a setting similar to Tanzania. Third, the attendance rate was much lower than what we had anticipated, which led us to part from some of our originally preplanned secondary outcomes and examine what affects attendance in more detail. However, we clearly specified which analyses and results were conducted post hoc so as to not hypothesize after the results were known (ie, avoid hypothesizing after the results are known [HARKing]) [27]. Furthermore, as the active posttrial follow-up could not be carried out over a short period, we cannot rule out that participants have been contaminated by this and, for example, have waited for a home visit and self-collected sample instead of having to go to the clinic. If this is the case, this could have had a negative effect on the attendance rate of the intervention and control group participants. Despite the fact that we pilot tested the text messages before starting the trial, it is plausible that the use of a health behavior theoretical framework and further pilot testing of the intervention and the text messaging portal could have addressed some of the study challenges.

Owing to the lack of blinding of participants, there is a risk of performance bias and risk of contamination, which could have affected the internal validity of the trial [28]. To preserve privacy, we did not personalize the text messages, and we excluded women who did not own a private mobile phone. However, this exclusion criterion affects the external validity of the trial, as the participants may not represent the target population. Despite our effort to ensure privacy for the study participants, we cannot guarantee that the participants found the messages confidential enough. If this was an issue, it could have affected the acceptability and effectiveness of the messages. Our explorative subgroup analysis indicated that HIV status may be a potential effect modifier. However, our trial was not dimensioned to assess differential effects across subgroups, and it is likely an artifact of the data and a false discovery rather than HIV status modifying the relationship between text messages and follow-up.

### Comparison With Prior Work

To our knowledge, this is the first trial from Africa that investigates if attendance to a health provider-initiated follow-up cervical cancer screening among HPV-positive women can be increased by the use of one-way text messages. No previous trials have addressed the follow-up of women who have tested HPV positive; hence, the actual comparability of our study results is limited. Nonetheless, the findings from the trial are somewhat in contrast to other cervical cancer one-way text message trials from East Africa. One trial from northern Tanzania found that one-way text messages increased the attendance rate among screening-naïve women (odds ratio [OR] 3.0, 95% CI 1.5-6.2) and that one-way text messages combined with an electronic voucher (eVoucher) increased it even further (OR 4.7, 95% CI 2.9-7.4). Still, the attendance rate was low, with 4.3% (12/281) of women attending in the control group, 12.9% (35/272) in the text message group, and 18.1% (54/298) in the text message plus eVoucher group, which indicates that one-way text messages did not manage to make the vast majority of women attend screening [13]. Furthermore, a trial from Kenya found that one-way text messages increased the attendance rate at a follow-up screening among women who had a normal pap smear at baseline (OR 8.0, 95% CI 4.7-13.7); 67.1% (96/143) of women attended in the intervention group and 20.3% (29/143) in the control group. However, the trial has been judged as a high-risk bias trial in a recent systematic review [10] and was published in a journal on Beall’s list of potential predatory publishers [29].

The outcome of our trial is highly relevant in a larger mHealth context and in relation to how to address the cervical cancer burden in East Africa. Rapid HPV testing is an area that has the potential to improve the prevention of the disease; however, this trial shows that implementation of rapid HPV testing leads to the challenge of providing proper follow-up of the women who test HPV positive. This is an issue that policy makers and global health clinicians should be aware of in relation to implementing rapid HPV testing as a primary screening method in future cervical cancer screening programs in Africa. Our posttrial follow-up strategy indicates that phone calls where nurses engage with the women and emphasize the importance of reattendance may be more effective than a one-way text message intervention. This finding is supported by a Malaysian trial on screening attendance, which found that one-way text messages had no effect, but phone calls did [12]. Furthermore, our trial shows that there is a potential for addressing the issue of nonattendance by implementing HPV self-sample testing at the home level. A qualitative interview study, which we conducted in the postintervention period among 15 women from the intervention group, showed that both behavioral and structural barriers affected screening reattendance, including fear of the gynecologic examination as well as transport and waiting time at the clinic [22]. HPV self-sample testing has the potential to overcome some of these barriers, and it has also proven to be an acceptable and effective method in other settings in Africa [30-33]. However, women who test positive to an HPV self-sample test would still require a form of visual inspection of the cervix to assess the features that would make it amenable or not. How best to implement rapid HPV tests, the overall care pathway for HPV-positive women and the potential of phone call reminders for cervical cancer follow-up are areas worth exploring in future studies in Africa.

### Conclusions

This study illustrates that the implementation of rapid HPV testing at cervical cancer screening clinics in Tanzania entails a number of challenges, including ensuring a proper follow-up of women who test HPV positive. One-way text messages had no effect on the attendance rate; however, it is plausible that phone calls or outreach services that involve HPV testing at home may be more promising methods than one-way text messages and repeat screenings at the clinic level. This should be explored in future studies.

## Appendix

Multimedia Appendix 1 CONSORT eHEALTH checklist (V 1.6.1).

## Abbreviations

CONCEPT: Comprehensive Cervical Cancer Prevention in Tanzania
eVoucher: electronic voucher
HPV: human papillomavirus
KCMC: Kilimanjaro Christian Medical Centre
mHealth: mobile health
OR: odds ratio
ORCI: Ocean Road Cancer Institute
RR: risk ratio
VIA: visual inspection with acetic acid
